# For-profit hospitals as anchor institutions in the United States: a study of organizational stability

**DOI:** 10.1186/s12913-021-07307-1

**Published:** 2021-12-11

**Authors:** Berkeley Franz, Cory E. Cronin, Vanessa Rodriguez, Kelly Choyke, Janet E. Simon, Maxwell T. Hall

**Affiliations:** 1 Department of Social Medicine, Heritage College of Osteopathic Medicine, Ohio University, Irvine Hall 210, Athens, OH 45701 USA; 2 Department of Social and Public Health, Ohio University, Grover Center W359, Athens, OH 45701 USA; 3 College of Applied Health Sciences and Wellness, Ohio Unitversity, Ohio Musculoskeletal and Neurological Design, Grover Center E150, Athens, OH 45701 USA

**Keywords:** Hospital, For-profit, Anchor institution, Closures, Mergers, Population health, Stability, Economics, Communities, Community health

## Abstract

**Background:**

Anchor institutions, by definition, have a long-term presence within their local communities, but it is uncertain as to whether for-profit hospitals meet this definition; most research on anchor institutions to date has been limited to nonprofit organizations such as hospitals and universities. Accordingly, this study aims to determine whether for-profit hospitals are stable enough to fulfill the role of anchor institutions through a long-term presence in communities which may help to stabilize local economies.

**Methods:**

This longitudinal study analyzes national, secondary data between 2008 and 2017 compiled from the Dartmouth Atlas of Health Care, the American Hospital Association Annual Survey, and County Health Rankings. We use descriptive statistics to calculate the number of closures and mergers of hospitals of different ownership type, as well as staffing levels. Using logistic regression, we also assessed whether for-profit hospitals had higher odds of closing and merging, controlling for both organization and community factors.

**Results:**

We found for-profit hospitals to be less stable than their public and nonprofit hospital counterparts, experiencing disproportionately more closures and mergers over time, with a multivariable analysis indicating a statistically significant difference. Furthermore, for-profit hospitals have fewer full-time employees relative to their size than hospitals of other ownership types, as well as lower total payroll expenditures.

**Conclusions:**

Study findings suggest that for-profit hospitals operate more efficiently in terms of expenses, but this also may translate into a lower level of economic contributions to the surrounding community through employment and purchasing initiatives. For-profit hospitals may also not have the stability required to serve as long-standing anchor institutions. Future studies should consider whether for-profit hospitals make other types of community investments to offset these deficits and whether policy changes can be employed to encourage anchor activities from local businesses such as hospitals.

## Background

Anchor institutions are large and stable institutions whose actions have an impact on the health, and social and economic strength, of their surrounding communities [1]. Moreover, anchor institutions have the ability to elevate population health by providing jobs and partnering with and investing in local businesses and community initiatives. Typically, scholars describe “meds and eds,” or medical and educational organizations, as anchor institutions given their likelihood to be rooted in their communities. This long-term presence, even in the midst of community change, helps to stabilize local economies [1–3]. Given federal requirements for non-profit hospitals to benefit their communities in exchange for tax exemption, most case studies of anchor hospitals have focused on this subset of hospitals and their efforts to elevate population health through targeted employment, purchasing, and health promotion [4]. Because of this limited focus, it’s not clear whether for-profit hospitals have similar institutional characteristics which are necessary to fill the role of anchor institution. An approach to engage for-profit hospitals, if successful, might contribute to improved population health and reduce disparities, especially in underserved U.S. communities where these institutions disproportionately operate [5–7].

By definition, anchor institutions have certain features that contribute to their positive impact on communities. These characteristics include longevity in communities and serving as large employers, taxpayers, and purchasers who can stabilize local economies. There is considerable evidence that large institutions, and hospitals in particular, are vital to the communities they inhabit and cause significant economic harm if they close or move from the community. Closures of anchor institutions, like any large employer, impact the local economy through job losses in the hospital itself as well as in related supply chain industries, and through a reduction in consumer service industries, such as stores, restaurants, and banks. The loss of disposable income in the community is especially consequential in communities where opportunities for employment are limited [8].

Existing research suggests that when hospitals close, communities not only lose economic resources, but a consistent supply of health care professionals and services [9]. Hospital closures are more likely to negatively affect the long-term economic prospects of a community when that hospital is the sole hospital in a community, such as in rural areas of the U.S. In these cases, the economic impact can be pronounced– with a lasting decline in per capita income and rise in unemployment [10]. Qualitative studies have documented resident perceptions of these closures, which include anger, resentment, and feelings of abandonment over the need to spend additional time and resources to gain access to health care services following a hospital closure [11].

Existing data suggest that hospital closures are not equally distributed and that certain environments and institutional structures shape these events and their likely impact on communities. For-profit hospitals are more likely to close than their nonprofit hospital counterparts [12–15]. Hospitals located in states that did not expand Medicaid also are at greater risk of closure, suggesting that hospitals may collapse under the weight of uncompensated care in environments where insurance coverage is lower [16]. Other hospital characteristics, beyond longevity in a community, also impact how much these institutions contribute to communities and may differ between for profit and nonprofit hospitals. For example, the ability of anchor hospitals to provide employment may be somewhat attenuated if hospitals systematically employ staff at different levels, relative to their size. Existing studies do not comment on whether for profit hospitals differ in this regard, but these and other organizational factors may contribute to their potential to function as an economic anchor.

## Objective

As we consider how hospitals of different ownership types may embody the characteristics of an anchor institution, it is critical to examine the stability of an organization and the extent to which that may affect an organization’s ability to support a community. Within this context, this study assesses the stability of hospital organizations in two ways: first through an analysis of closures and mergers and second through an assessment of regular staff employed. These factors will provide an understanding of the extent to which for profit hospital organizations have the potential to serve as effective anchor institutions for their communities. In doing so, this study will be the first to utilize over 10 years of data on hospitals in the U.S. to assess the likelihood of closures and mergers and to track long-term employment trends. These findings will provide important insight into whether for-profit hospitals are as likely as other hospitals to serve as anchor institutions in U.S. communities.

## Methods

This longitudinal study was conducted with data from Dartmouth Atlas of Health Care linked with the American Hospital Association (AHA) Annual Survey and County Health Rankings for the years 2008–2017 [17, 18]. These data are ideally suited to the research question because of the comprehensive nature of the hospital data, which include both recent and historical information. The AHA dataset, in particular, is the most complete collection of data on the U.S. hospital population, including for-profit hospitals [19].

In analyzing these data, we utilize a national dataset of general-medical hospitals. While AHA data include all types of hospitals, those that do not fit the description of a general medical hospital were removed for the purposes of this study. We also removed all federal hospitals, considering that their federal support creates different expectations of stability and their purposes are often focused on particular populations.

The unit of analysis for the multivariable model is hospital year, with hospitals repeated in the years 2008, 2009, 2010, 2011, 2012, 2013, 2014, 2015, 2016 and 2017. This resulted in a sample of 46,178 hospital years. We merged AHA data with data from County Health Rankings and removed hospital years with missing data via listwise deletion which resulted in our final analytic sample of 44,128 hospital years. The variable that was most commonly the cause of missing data was the poor/fair health rating from County Health Rankings. To draw contrast in hospital staffing trends by ownership type, an additional descriptive analysis was conducted between the years 2008 and 2017, allowing us to consider changes that had occurred between those two points in time.

### Study measures

The multivariable analysis considers two dependent variables: whether the hospital closed during the studied time period and whether the hospital merged with another organization during the studied time period. A merger may include an individual hospital joining a system or changing systems, or a system-level merger for a hospital that was already part of a system.

Independent measures for this study include both hospital characteristics and characteristics of the hospital’s county. Hospital measures include ownership (for-profit compared to nonprofit/public); number of beds (50–199, 200–399, and 400 or more as compared to the reference group of 0–49) and system membership (coded 1 if a hospital system member, 0 if not). County characteristics include the percent of residents reporting poor or fair health; the total number of hospital beds per 1000 county residents; rural classification; and region of the country (Northwest, Midwest, or West, as compared to the South for reference). Hospital staffing variables used in the descriptive analysis for the years 2008 and 2017 include full time equivalents (FTEs) per bed; registerd nurse (RN) FTEs per bed; payroll per bed; and employee benefits per bed.

### Statistical analyses

All analyses were completed in 2020 using STATA 16 [20]. Because of the longitudinal time series nature of this data, we utilized a panel data design specifying a unique ID and year for each hospital, resulting in hospital-years as the unit of analysis. However, for the hospital closures, results showed this methodology not to be appropriate (rho = 0.0001), and the likelihood test was not significant (chibar2(01) = 0.001, prob. = 0.496) indicating that the panel-level variance component (years) is unimportant, and the panel estimator is no different than the pooled estimator. Therefore, we ultimately only used this method for the merger analysis. For the panel data analysis, we calculated rho and sigma. Rho indicates the proportion of variance explained by the group level. When rho is zero, the panel-level variance component is unimportant, and the panel estimator is no different from the pooled estimator. Sigma indicates the panel-level standard deviation. Akaike Information Criterion (AIC) and Bayesian Information Criterion (BIC) were calculated for each regression model to assess the model fit.

The descriptive portion of the analysis included frequency, percent, and mean (when appropriate) for each of the study variables. We considered descriptive statistics for the entire sample, as well as for the for-profit subset separately. In the multivariable analysis, we employed logistic regression due to the dichotomous nature of the two dependent variables. The multivariable model considers both organization and county characteristics as control variables.

## Results

**Fig. 1:**
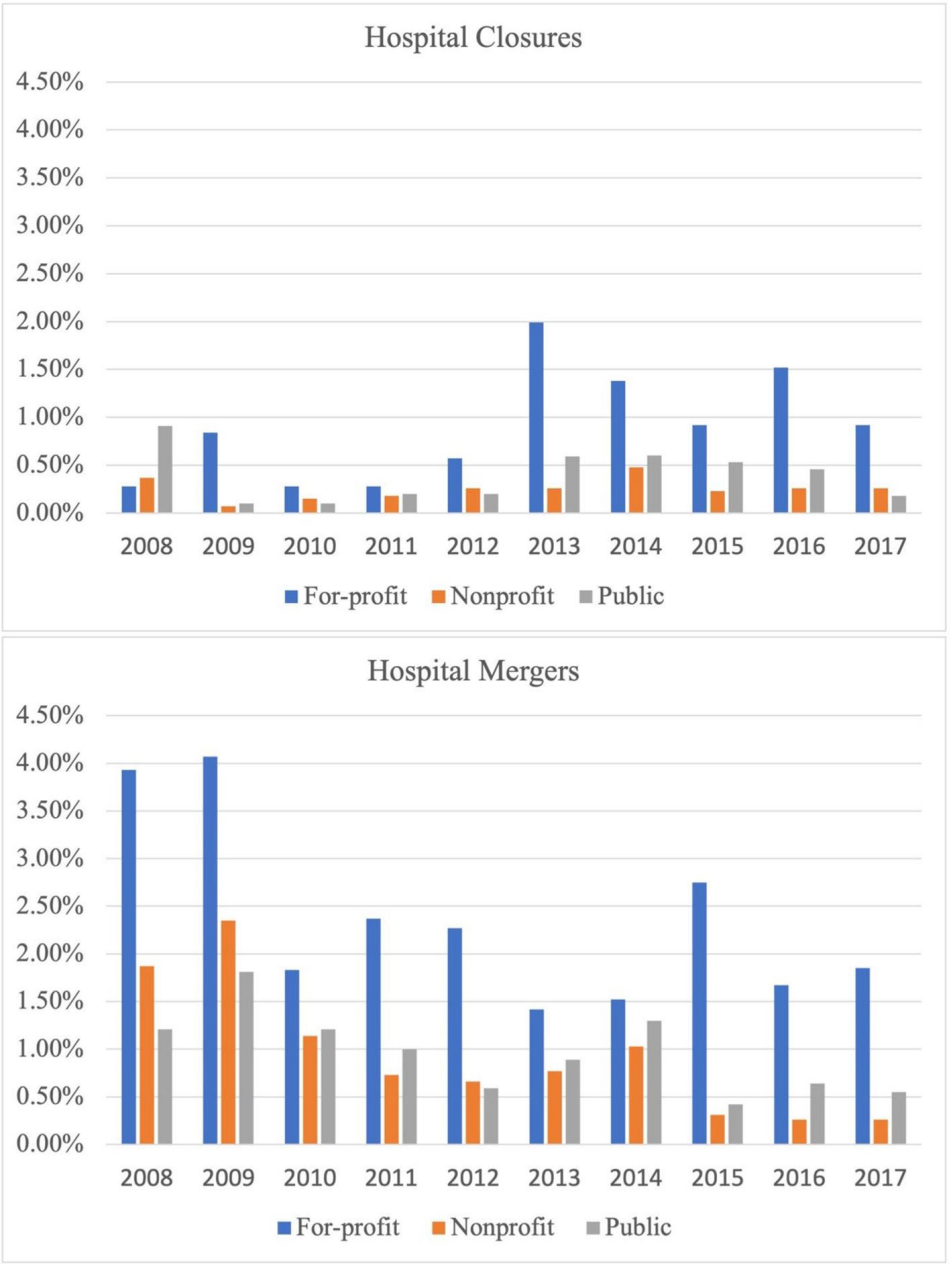
Percent of Each Hospital Type Experiencing Organizational Change

Descriptive statistics indicate that 16% of the hospital-years in the sample, or 6948 total, represent for-profit ownership. The for-profit subsample of hospitals tends to be characterized by smaller organizations (fewer hospital beds), in areas with more health needs, as measured by the percentage of residents in poor or fair health, and more concentrated in the Southern region. Descriptive statistics also indicate that for-profit hospitals are overrepresented in the number of hospital closures and mergers overall during the studied time period (see Table 1) and across time (Fig. 1).

**Table 1:** Descriptive Statistics of Hospital and County Characteristics, 2008–2017 (*N* = 44,128 hospital-year observations)

	Total Sample		For-profit Only	
Hospital Characteristics	Frequency	%	Frequency	%
**Ownership**				
For-profit	6948	15.75	6948	100
Nonprofit	27,060	61.32		
Public	10,120	22.93		
**Number of Beds**				
0–49	12,488	28.30	1674	24.09
50–199	18,460	41.83	3685	53.04
200–399	8911	20.19	1312	18.88
> 400	4269	9.67	277	3.99
**Closed**	169	.38	62	.89
**Transit**	517	1.17	165	2.37
**System Membership**	26,939	61.05	5260	75.71
**Rural**	17,590	39.86	1810	26.05
**Region**				
Northeast	5740	13.01	207	2.98
Midwest	13,193	29.90	705	10.15
West	8793	19.93	1518	21.85
South	16,402	37.17	4518	65.03
**Year**				
2008	4430	10.04	712	10.25
2009	4430	10.04	712	10.25
2010	4430	10.04	712	10.25
2011	4448	10.08	718	10.33
2012	4441	10.06	706	10.16
2013	4445	10.07	704	10.13
2014	4454	10.09	724	10.42
2015	4215	9.55	654	9.41
2016	4429	10.04	657	9.46
2017	4406	9.98	649	9.34
**County Characteristics**	**N**	**Mean (SD)**	**N**	**Mean (SD)**
**Percent Reporting Poor/Fair Health**	44,128	16.41 (4.98)	6948	18.29 (4.87)
**County Beds / 1000 Population**	44,128	5.58 (7.06)	6948	4.86 (5.17)

For-profit hospitals also have fewer full-time positions relative to their size than hospitals of other ownership types, both for total employees (for-profit hospitals 5.39 FTEs per bed compared to 6.56 FTEs per bed of other hospital types in 2017) and for clinical roles (1.82 compared 1.76 for RN specific FTEs per bed). Payroll per bed is also significantly lower (*P* < .05) for for-profit hospitals, as are employee benefit costs. When looking at both the beginning point and the ending point of our study, gaps between hospital ownership types occur at both points in time, though the gap in RN FTEs does close to some extent in that time period (see Table 2).

**Table 2:** Mean Comparison of Hospital Bed Characteristics by Ownership, 2008 and 2017

	2008				2017			
	For-profit		Other ownership		For-profit		Other ownership	
	N	Mean	N	Mean	N	Mean	N	Mean
**FTE per bed**^**a**^	629	4.41*	3541	5.79	629	5.39*	3712	6.56
**RNs per bed**	629	1.34*	3541	1.41	629	1.82	3712	1.76
**Payroll per bed**	629	$220,353*	3541	$302,499	629	$339,260*	3711	$428,565
**Benefits per bed**	629	$48,666*	3541	$78,471	629	$75,187*	3712	$113,946

^a^ Boldface indicates statistical significance using two-tailed independent samples T-Test (*p < .05)

The multivariable model predicting hospital closures found a positive significant association for for-profit ownership and communities reporting poorer health (odds ratio [OR] = 1.06, 95% Confidence Interval (CI) = 1.03–1.10, *P* < .001) (Table 3). This indicates that hospitals that are for-profit and communities reporting poorer health had higher odds of closure. The following variables had a significant negative association with hospital closures: hospitals with 200–399 beds (OR = 0.517, 95% CI = .297–.901, *P* = .020) or more than 399 beds (OR = 0.181, 95% CI = .0551–.596, *P* = .005), system member hospitals (OR = 0.084, 95% CI = .0530–.132, *P* < .001), rural location (OR = 0.442, 95% CI = .306–.640, P < .001), and the earliest years of the study 2009 (OR = 0.424, 95% CI = .193–.929, *P* = .032) and 2010 (OR = 0.329, 95% CI = .139–.778, *P* = .011). This indicates that hospitals with larger numbers of beds, system member hospitals, and rural location are protective factors for hospital closure.

**Table 3:** Logistic Regression of the Relationship Between Hospital Closures and Hospital/Community Characteristics (N = 44,128 hospital-year observations)

		95% CI	
	OR	Low	High
**Non-profit/Public**	Reference	Reference	Reference
**For-profit**^**a**^	3.187***	2.226	4.562
**Beds = 0–49**	Reference	Reference	Reference
**Beds = 50–199**	0.947	0.671	1.336
**Beds = 200–399**	0.517*	0.297	0.901
**Beds > 400**	0.181**	0.055	0.596
**System Membership**	0.084***	0.053	0.132
**Percent Reporting Poor/Fair Health**	1.061***	1.027	1.097
**Rural County**	0.442***	0.306	0.640
**County Beds / 1000 Population**	1.020	0.999	1.042
**Region – South**	Reference	Reference	Reference
**Region - Northeast**	1.304	0.759	2.240
**Region - Midwest**	0.676	0.416	1.098
**Region – West**	0.653	0.414	1.029
**Year – 2008**	Reference	Reference	Reference
**Year - 2009**	0.424*	0.193	0.929
**Year - 2010**	0.329*	0.139	0.778
**Year - 2011**	0.489	0.218	1.098
**Year - 2012**	0.717	0.348	1.476
**Year - 2013**	1.525	0.832	2.798
**Year - 2014**	1.572	0.864	2.860
**Year - 2015**	1.143	0.582	2.243
**Year - 2016**	1.458	0.772	2.752
**Year – 2017**	1.002	0.499	2.011
**AIC**	1936.299		
**BIC**	2118.89		

^a^Boldface indicates statistical significance (***p < .001; **p < .01; *p < .05)

The likelihood ratio test for the proportion of variance attributable to years was significant rho = 0.83, chibar2(01) =1496.51, prob. = 0.001. The multivariable model predicting hospital mergers found a positive significant association (OR = 7.51, 95% CI = 3.97–14.21, *P* < .001) for for-profit ownership controlling for hospital and community-level factors. Communities reporting poorer health, counties with more hospital beds, and the Northeast region also had higher odds of merging (see Table 4). The following variables had a negative association with hospital mergers: hospitals with 200–399 beds or more than 399 beds, system member hospitals, and rural location. This indicates that hospitals with larger numbers of beds, system member hospitals, and rural location may be protective factors for hospital merger.

**Table 4:** Logistic Regression of the Relationship Between Hospital Mergers and Hospital/Community Characteristics (N = 44,128 hospital-year observations)

		95% CI	
	OR	Low	High
**Non-profit/Public**	Reference	Reference	Reference
**For-profit**^a^	7.513***	3.971	14.214
**Beds = 0–49**	Reference	Reference	Reference
**Beds = 50–199**	1.196	0.702	2.038
**Beds = 200–399**	0.310**	0.135	0.712
**Beds > 400**	0.011**	0.001	0.166
**System Membership**	0.026***	0.012	0.053
**Percent Reporting Poor/Fair Health**	1.071*	1.025	1.120
**Rural County**	0.306***	0.178	0.525
**County Beds / 1000 Population**	1.067***	1.051	1.083
**Region – South**	Reference	Reference	Reference
**Region - Northeast**	2.747*	1.254	6.017
**Region - Midwest**	0.550	0.271	1.116
**Region – West**	1.043	0.547	1.989
**sigma**	4.014	3.760	4.285
**rho**	0.830	0.811	0.848
**AIC**	3528.892		
**BIC**	3641.925		

^a^a Boldface indicates statistical significance (***p < .001; **p < .01; *p < .05)

*AIC* Akaike Information Criteria

*BIC* Bayesian Information Criteria

## Discussion

City planners and policy experts are increasingly looking to for-profit businesses to serve as community-engaged institutions who stabilize the economic base of U.S. communities and bolster health and well-being [21, 22]. For-profit hospitals may be one example of businesses being successfully leveraged to improve local communities, but the current study findings suggest that one challenge to the prospect of for-profit hospitals serving as anchor institutions may be their stability and longevity in the communities they serve. For-profits are more likely than their counterparts to close or to merge, which indicates that, as an organizational type, they are less reliable to provide economic support over time to a community. Closures are a clear example of a way in which an organization may exit a community, taking with it a substantial number of jobs and other means of injecting money into a local economy. Beyond closures, this is important to consider in the case of mergers as well, since a merging hospital may find itself with a different identity or mission, potentially weakening its established community ties, or even with a reduced workforce. At the same time, for-profit hospitals tend to exist in areas with greater health needs [23]. These areas also face greater numbers of hospital closures and mergers overall, as our findings show. This combination of factors indicates that for-profit hospitals hold critical potential for strengthening local economies but may be limited by organizational characteristics that make them less stable in communities where there is also greater need.

It is perhaps unsurprising that for-profit hospitals, which by their nature have the purpose of generating revenue for shareholders, are more inclined to pursue business strategies such as closures or mergers when operation in a community no longer becomes financially advantageous. Yet, such strategies may be in conflict with a mission of serving a community’s well-being, forcing for-profit hospitals to reconcile their business decisions as a for-profit organization with the purpose they serve as a hospital. This may be further complicated by a decentralized decision-making structure that is common with for-profit hospitals that exist as part of a system; in such systems, headquarters that exist elsewhere and the executives who work there may not be plugged into the needs of a local community, making the consequences of an organization’s business strategies less apparent.

Departures of hospitals from rural communities have the potential to hit particularly hard in this way. One finding of our analysis portrays hospitals to be more stable in rural communities, which appears to counter recent literature on the topic. Considering this, we completed a post-hoc analysis to consider rural closure trends, which provide a clearer narrative [9]. While rural closures were less likely to occur when looking at the studied time period as a whole due to fewer occurrences as compared to non-rural communities in the early years of the study, we can see a clear trend in rural hospital closures increasing over time. For example, in 2010, .1% of rural hospitals closed, compared to .2% of nonrural; in 2016, .8% of rural hospitals closed, compared to .3% of nonrural (see Fig. 1). This is a phenomenon worth continued examination and study, particularly given the challenges it presents for rural communities both in regard to health and economic well-being.

Beyond institutional stability, another defining characteristic of anchor institutions is their propensity to employ large numbers of people [2, 24]. Again, study findings indicate that this is an area where a for-profit organization’s business strategies may result in less economic benefit for the community. Likely due to their emphasis on fiscal efficiency, for-profit hospitals employ fewer people than nonprofit hospitals of a comparable size. It is interesting that the employment numbers are more similar for clinical roles, which may point to accreditation or licensure standards that are applicable to all hospitals across ownership status or quality of care standards all hospitals would consider [25–27]. On the other hand, all types of hospitals may be facing consequences of a national nursing shortage and therefore find themselves unable to staff beyond certain levels [28].

Despite less stability and lower employment levels, for-profit businesses, including hospitals contribute to the local tax base which may support the economic health of their communities in ways not shared by private non-profit and public institutions. In both urban and rural communities where major institutions have left, for-profit hospitals may contribute to a shrinking tax base and provide critical support for community health and well-being. It is not clear, however, if these contributions are enough to offset other characteristics, such as the propensity to close, when weighing their economic contributions to U.S. communities. For-profit hospitals, like other businesses, may also make other contributions to their communities which are not measured in this study, including through health promotion and community development partnerships. This may include wellness programs among employees or extend to the community at large [29]. Indeed, the emphasis on social in addition to fiscal responsibility, is evident among some for-profit businesses and there may be potential to leverage such investments to improve the health of communities [21, 30]. Because of their size and influence, for-profit organizations, including hospitals, should not be discounted as potential anchor institutions, but more studies are needed to understand the extent and scope of their contributions in U.S. communities.

A recognition that for-profit hospitals have the potential to serve as anchor institutions should not be confused with an assumption that all for-profit hospitals have the desire or even willingness to take on this role and the greater community responsibility it may represent. Future studies are necessary to understand how for-profit hospitals define their role in population health improvement, including through traditional anchor activities like employment and procurement or through direct investments and community partnerships. Recent changes in reimbursement may encourage moving toward value from traditional fee-for-service models [31, 32], but whether these changes are influential in regard to anchor activities is still unknown. Policymakers should consider the potential role that for-profit hospitals may play in population health initiatives and what policies may activate investments in this area in the absence of formal community benefit responsibilities.

## Limitations

There are several limitations that must be weighed against the novel contributions of this study. The first is that the data are self-reported; there is the possibility that hospitals completing the annual AHA survey interpret questions differently. It is also important to note that closures are a rare event, which means that we are assessing variation among a very small sample of hospitals. Due to our reliance on secondary, quantitative data, we also have limited context to understand why organizational characteristics differ by hospital type. We are also unable to evaluate outcomes of these events. It is possible that a merger may provide added resources to a community, or that a closure of a hospital does not mean a departure entirely of a health care organization’s presence in a neighborhood (e.g., a hospital ceased providing inpatient services, but remains as an outpatient care provider). In both of these scenarios, mixed methods or qualitative research studies would provide more insight into the impact of these changes on the well-being of community residents. These methods would also allow for a more nuanced understanding of organizational decision-making and the link between ownership type and key hospital characteristics.

## Conclusion

For profit hospitals are not often considered as anchors, despite serving as key institutions in their communities. For profit hospitals, unlike their nonprofit counterparts, contribute to the local tax base and may stabilize their communities in ways that researchers have not fully appreciated. Still, for profit hospitals have characteristics that set them apart from other hospitals and which may limit their ability to provide long term support for community health and economic stability. Recognizing the potential contributions of these institutions is critical to understanding what communities stand to gain and lose when hospitals participate in population health improvement. These data will help policymakers leverage support for expanded incentives for explicitly adopting an anchor framework for population health improvement.

## Data Availability

The County Health Rankings data analyzed during the current study are publicly available at https://www.countyhealthrankings.org/. The Dartmouth Atlas of Health Care data analyzed during the current study are publicly available at https://www.dartmouthatlas.org/. Additional data analyzed during the current study are available from the American Hospital Association Annual Survey but restrictions apply to the availability of these data, which were used under license, and so are not publicly available. Data are however from the authors upon reasonable request and with the permission of the American Hospital Association.

## Abbreviations

AIC: Akaike’s Information Criterion
AHA: American Hospital Association
BIC: Bayesian Information Criterion
CI: Confidence Interval
FTE: Full Time Equivalent
OR: Odds Ratio
RN: Registered Nurse
